# What Are the Potential Benefits of Using Bacteriophages in Periodontal Therapy?

**DOI:** 10.3390/antibiotics11040446

**Published:** 2022-03-25

**Authors:** Jan Kowalski, Renata Górska, Martyna Cieślik, Andrzej Górski, Ewa Jończyk-Matysiak

**Affiliations:** 1 Department of Periodontology and Oral Diseases, Medical University of Warsaw, 02-097 Warsaw, Poland; jan.kowalski@wum.edu.pl (J.K.); r_gorska@interia.pl (R.G.); 2 Bacteriophage Laboratory, Hirszfeld Institute of Immunology and Experimental Therapy, Polish Academy of Sciences, 53-114 Wrocław, Poland; martyna.cieslik@hirszfeld.pl (M.C.); andrzej.gorski@hirszfeld.pl (A.G.); 3 Phage Therapy Unit, Hirszfeld Institute of Immunology and Experimental Therapy, Polish Academy of Sciences, 53-114 Wrocław, Poland; 4 Infant Jesus Hospital, The Medical University of Warsaw, 02-006 Warsaw, Poland

**Keywords:** bacteriophage, periodontitis, antibiotic resistance, biofilm, red complex

## Abstract

Periodontitis, which may result in tooth loss, constitutes both a serious medical and social problem. This pathology, if not treated, can contribute to the development of, among others, pancreatic cancer, cardiovascular diseases or Alzheimer’s disease. The available treatment methods are expensive but not always fully effective. For this reason, the search for and isolation of bacteriophages specific to bacterial strains causing periodontitis seems to be a great opportunity to target persistent colonization by bacterial pathogens and lower the use of antibiotics consequently limiting further development of antibiotic resistance. Furthermore, antimicrobial resistance (AMR) constitutes a growing challenge in periodontal therapy as resistant pathogens may be isolated from more than 70% of patients with periodontitis. The aim of this review is to present the perspective of phage application in the prevention and/or treatment of periodontitis alongside its complicated multifactorial aetiology and emphasize the challenges connecting composition and application of effective phage preparation.

## 1. Introduction

Chronic inflammation of periodontal tissues (periodontitis) and a similar disease occurring around dental implants (peri-implantitis) are major concerns in modern dentistry. Both of these diseases, though not life-threatening, lead to the deterioration of the patient’s health and quality of life. They are very common and multifactorial diseases in which both the microbial component and host response play a crucial role. There is evidence that the occurrence of chronic periodontitis may correspond with, among others, Alzheimer’s disease, stroke, and a higher risk of preterm low birth-weight infants, type 2 diabetes mellitus or arteriosclerosis [1,2,3,4,5].

Conventional methods of periodontal disease treatment (dental biofilm control, improving the effectiveness of oral hygiene, adjunctive therapies for gingival inflammation, mechanical plaque removal, using local and/or systemic antimicrobial [6]) are not fully effective. Therefore, bacteriophages as a natural component of both the environment and the human body, pose as a type of effective and highly targeted therapy (because of their specificity), their potential use in combating periodontitis seems to be rational and justified. Isolation of new phages is a relatively inexpensive and fast process [7,8] compared to research on the development and introduction of new antimicrobials, and their use is less expensive than conventional antibiotic treatment. Furthermore, the use of phages does not cause serious side effects or disturb the composition of the natural microbiota [9,10] which are observed with the use of antibiotics [11]. Using antibiotics may cause the acquisition of resistance of bacterial strain for these antimicrobial agents. According to the World Health Organization (WHO), antimicrobial resistance is on the list of the ten most serious threats to public health [12]. In view of this, it has been proven that phages can be active against antibiotic resistant strains. For example, therapeutic application of phage ABP1 rescued mice infected with *Acinetobacter baumannii* exhibiting pan-drug resistance [13] which confirms the effectiveness of phage therapy. Although no reports of using phage therapy in the treatment of periodontitis have been described so far, at the moment, there is no effective available therapeutic agent with both preventive and therapeutic effects, and applying bacteriophages has a good chance to effectively fight against periodontal disease and should fill the niche in this area.

## 2. Epidemiology of Periodontal Diseases

Approximately 11% of the world’s adult population suffers from advanced forms of periodontitis requiring specialist intervention [14]. Age-standardized incidence of this form reached 701 cases per 100,000 person-years in 2010 [15]. This number may vary depending on numerous factors, including the nation’s hygiene self-awareness and quality of dental service [16]. Research conducted in the largest cities in Poland in 2012 revealed that 16% of the adult population was affected by a severe form of periodontitis [17]. German researchers estimated the total and annual treatment costs per individual patient of advanced periodontitis as EUR 7154 and EUR 437, respectively [18]. An aging population with more preserved teeth and the increased popularity of implant treatment, often resulting in the loosening of strict initial exclusion criteria, will surely lead to increased incidences of both periodontitis and peri-implantitis. Dental implants which replace missing teeth are commonly applied [19]. It is estimated that worldwide each year as many as 12 million implants are placed [20].

## 3. Etiopathogenesis of Periodontitis

Periodontitis is a chronic immune-inflammatory disease in which microbiological determinants, as well as host individual (genetic and epigenetic) factors, are crucial. The degeneration of tissues supporting teeth/implants (bone and tissue loss) results from the non-specific inflammatory response of the organism to periopathogens [21]. There is no single specific bacterial species responsible for this process, although one can distinguish the group of Gram-negative anaerobic rods associated with disease sites [22]. Along with the development of metagenomics, the issue of the local participation of bacteria in the pathogenesis of periodontitis turned out to be much more complicated. The presently established etiological model acknowledges the almost constant presence of bacteria on the oral tissues. In healthy individuals, the dental biofilm is immature and is continuously reduced by means of oral hygiene. It is mostly harmless for the host and is defined as a eubiotic biofilm. It consists predominantly of Gram-positive staphylococci and streptococci, and other bacteria formerly included in so-called yellow and purple clusters or complexes [23]. While the concept of bacterial complexes is regarded as obsolete, it helps in understanding the nature of biofilm maturation and the necessity of the appearance of certain bacteria before other ones can have the chance to survive in the oral environment. Periodontitis, on the other hand, is associated with dysbiotic biofilm. It is still under discussion whether dysbiosis enhances inflammation and disease progression, or vice versa [24].

Bacteria involved in the development of periodontitis are mainly classified into 17 species belonging to: *Bacteroidetes*, *Saccharibacteria*, *Firmicutes*, *Proteobacteria*, *Spirochaetes* and *Synergistetes*. Those that are suggested to be related to the most active disease are: *Porphyromonas gingivalis*, *Treponema denticola*, *Tannerella forsythia* [25].

There are many factors contributing to the pathogenicity of periodontitis-associated bacteria. Microorganisms, both pathogenic and non-pathogenic, need various factors that favor their adhesion in the process of colonization of the oral cavity. These include the affinity of bacterial adhesins (such as lipopolysaccharide (LPS), fimbriae or capsule) for the receptors on cells in the host’s oral cavity [25]. Below and in Table 1, we describe the factors that are particularly important for the indicated types of bacteria. These factors increase the virulence and spreadability of bacteria, as well as directly damage oral tissues (enzymes like hyaluronidase and beta-glucuronidase, which mainly applies to *Staphylococcus aureus*, *Streptococcus pyogenes* and *Clostridium histolyticum* [25]) and interfere in the immune response. For example, dentilisin from *T. denticola* promotes the production of pro-inflammatory cytokines (tumor necrosis factor-α (TNF-α), interleukin-1β (IL-1β) and interleukin-6 (IL-6)) and then degrades them, which can cause long-lasting infections [26]. Citrullinated proteins, arising under the influence of peptidyl-arginine deiminase (PAD) from *P. gingivalis*, are powerful antigens, which may lead to the development of autoimmune diseases associated with chronic periodontitis [27]. Toxic factors produced by *P. gingivalis*, like LPS, pili and gingipains (cysteine proteases), not only damage tissue directly, but also interfere with the host’s immune response by influencing the immune cells in the oral cavity through various TLRs (Toll-like receptors) causing secondary damage [28]. Interestingly, phosphorylation turns out to be crucial in the processing of *P. gingivalis* virulence factors [29]. Some bacteria, including *P. gingivalis*, may evade immune response, avoid phagocytosis by macrophages [30]. Moreover, both the bacterial outer membrane proteins and LPS derived from them may have a strong influence on the disturbance of the secretion of antimicrobial peptides in the oral cavity. One such example is the overexpression of human β-defensin 2 (hBD-2) by the oral epithelium, resulting in an exacerbation of inflammation [31]. Hydrogen sulfide produced by bacteria (*P. gingivalis*, *T. forsythia*, *T. denticola* or *F. nucleatum*) induces an immune response and the release of pro-inflammatory cytokines, such as IL-1β and IL-18 by monocytes, as well as apoptosis of fibroblast cells in the gingiva. Moreover, the ability of bacteria (such as *Streptococcus* spp. or *Fusobacterium nucleatum*) to form biofilm is of great importance in the development of periodontitis, and cooperation in the biofilm structure by various species of bacteria causes the availability of methods of combating them to be limited [32].

The virulence factors mentioned above induce an immune response and the susceptibility to chronic periodontal disease is an effect of inflammation resulting from the interaction between environmental and host genetic factors [33,34].

Macrophages, neutrophils and other cells are stimulated to produce cytokines, an unbalanced production which causes periodontal tissue damage [35]. TNF-α, IL-1β, (interleukin-8 (IL-8), prostaglandin E_2_ (PG E_2_) are, among others, secreted pro-inflammatory cytokines. IL-1, IL-8 and TNF-α are responsible for the promotion of neutrophils to the site of inflammation, whereas IL-1 was found to have the ability to enhance expression of the receptor activator of nuclear factor-kappa B (NF-κB) ligand (RANKL) localized on osteoblast and T helper cells which upregulate osteoclasts maturation and result in alveolar bone loss [36,37]. During periodontal disease, an increase in the level of matrix metalloproteinases (MMPs) responsible for the destruction in collagen fibers in periodontal tissue has been observed [37].

Interestingly, a correlation between polymorphisms in cytokine genes and susceptibility to chronic periodontitis have been indicated [33,38]. These authors presented that single nucleotide polymorphisms in the IL-1α, IL-1β, IL1RN, IL-6, IL-10, TNF-α, transforming growth factor β1 (TGF-β1), interferon γ (IFN-γ) and vitamin D receptor (VDR) may be associated with susceptibility to chronic periodontitis. Similarly, Liu and Li (2022), based on available published data, indicated that the polymorphism of IL-1β may be used as a biomarker in risk of periodontitis assessment [39].

However, the aetiology of periodontitis does not only rely on the presence or absence of these species, because it has been proven that these bacteria are also detectable in healthy individuals [40]. It is rather the change of the proportions of given bacteria, as well as a change in their properties [41]. Such change in activity is the result of growth and differentiation of the complex bacterial macrostructure, called dental biofilm. Several hundred bacterial species are involved in its existence (one estimates that in the oral cavity, the number of species exceeds 700 (1000 if fungi, viruses and protozoa are included), and in one milliliter of saliva there is more than 10^9^ bacteria [42]. Under normal conditions, the oral cavity microbiota’s composition is in a state of balance. Bacteria constantly present in the oral cavity, play a role in maintaining oral and systemic health [43]. Deterioration of oral hygiene, smoking, genetic predispositions, diabetes—disturbing proper immune response, caries, retentive spots or hyposalivation—creating favorable conditions for colonization, may be factors that influence the initiation and progress of the disease [44].

**Table 1:** Virulence factors that are particularly important for the indicated types of bacteria.

Bacterial Species	Virulence Factor	Effect
*Porphyromonas gingivalis*	peptidyl-arginine deiminase (PAD) [27]	adapting bacteria to survive in an acidic environment
	gingipains (cysteine proteases) [28]	tissue damage; interference in human immune system
	internalin protein InlJ [45]	biofilm development
*Treponema denticola*	flagellin, a component of flagella [27]	ability to move; stimulating the immune system
	type III secretory system [27]	extracellular secretion of other virulence factors (mainly proteins)
	dentilisin (protease) [26]	stimulation of production followed by degradation of IL-1β, IL-6 and TNF-α
	leucine-rich repeat LrrA protein [46]	binding to and penetration of human epithelial cells; coaggregation with *T. forsythia*
*Tannerella forsythia*	leucine-rich repeat BspA protein [47]	biofilm development; coaggregation with *P. gingivalis*
	karilysin [48]	dissemination of TNF-α from macrophages; degradation of antimicrobial peptides
*Aggregatibacter actinomycetemcomitans*	adhesins [49]	binding to specific receptors in the oral cavity
	invasins [49]	penetration of bacteria into the host cells
	leukotoxin LtxA [50]	cells lysis; degranulation of human leukocytes

## 4. Difficulties with Treatment

The complex nature of the biofilm and resistance to chemical agents in the case of periodontal disease require a mechanical treatment which includes its (supra- and subgingival) nonsurgical removal and surgical correction of destroyed tissues (often including regeneration techniques and materials). Chemical treatment is based on topical application of antimicrobial agents (chlorhexidine, povidone-iodine, essential oils or hydrogen peroxide) used in mouth rinses or dentifrices, as well as antibiotic therapy. The latter has been widely used in complex cases of advanced tissue destruction. It is postulated that local (topical) administration of drugs (including antibiotics), as adjuvants in the treatment of periodontal diseases may have more advantages than systemic antibiotic therapy [51,52,53]. Despite many positive effects, this way of administering antimicrobial agents requires appropriate delivery and release mechanisms. The importance of vehicles with the proven sustained release is particularly emphasized [54]. A promising formulation of minocycline (in the form of a lipid complex) using a biodegradable polymer has recently been described and has shown good effects in combating bacterial biofilm in vitro [55]. Furthermore, in the case of therapeutic bacteriophages, various new and interesting ideas for their local delivery to the target site are described [56], but to our knowledge, there are no studies in this area on bacteria associated with periodontitis.

Recently, however, the major issue of antibiotic resistance has been raised, and recent recommendations of major periodontal associations do not recommend general administration of the antibiotics, fearing further development of drug-resistant bacterial strains [6,57,58]. Recommendations correspond to treatment protocols for the periodontitis stage I–III. However, for periodontitis stage IV in development, necrotic disease is not the subject of the recommendation. The most commonly characterized antibiotics in the treatment of periodontitis are drugs with various effects from eight groups: amoxicillin, ampicillin, tetracycline, minocycline, doxycycline, erythromycin, clindamycin and metronidazole, which are selected due to, inter alia, present pathogen, patient’s age, existing drug allergies, kidney function [59].

An important issue that determines therapeutic failures in antibiotic treatment is the fact that bacteria acquire increasing resistance to antimicrobial reagents. Bacteria that produce beta-lactamases—enzymes responsible for the hydrolysis of beta-lactamase antibiotics, including widely used penicillins, cephalosporins, monobactams or carbapenems—are found in most patients (68%) suffering from refractory periodontitis. The main species associated with periodontitis and showing this type of resistance is *Prevotella* [60]. The results of other studies indicate the presence of resistant pathogens isolated from inflamed periodontal pockets in more than 70% of patients with confirmed chronic periodontitis, while the most resistant strains were also *Prevotella* (*P. intermedia* or *P. nigrescens*), as well as *A. actinomycetemcomitans* and *Streptococcus constellatus*. In vitro studies have shown a lack in the susceptibility of bacterial strains mainly to doxycycline, but also to amoxicillin, clindamycin or metronidazole [61]. Recent studies on samples from German dental practices and hospitals have shown that *Staphylococcus* and *Streptococcus* spp. may be the prevalent pathogens associated with odontogenic infections, which show significant resistance to a broad spectrum of antibiotics, with more than 17% of strains not being susceptible to macrolide and clindamycin. Interestingly, more resistant bacteria were isolated from patients requiring hospital care than those using the dental clinic [62]. There are also studies showing that among bacteria (such as *P. gingivalis*, *P. intermedia*, and *P. nigrescens*) isolated from oral samples taken from children, nearly half of them contained tetracycline and/or erythromycin resistance genes (*tet*(*Q*) and *erm*(*F*), respectively) [63]. On the other hand, bacteria such as *A. actinomycetemcomitans*, *P. gingivalis* and *T. forsythia* often present resistance to amoxicillin, azithromycin and metronidazole, while studies using moxifloxacin have shown effective bactericidal activity [64]. Moreover, there are also reports highlighting the growing problem of drug resistance among the bacteria that cause gum disease [65]. To keep up against bacterial resistance (especially new and more sophisticated mechanisms), new antibacterials should soon be introduced and available to patients suffering from periodontal diseases. Unfortunately, in the last 30 years, no new antibiotic groups have been developed [66]. This points to the inevitability of losing antibiotics as both a useful and effective tool in treatment, and urges searching for novel therapeutic approaches [67]. The analysis of antibiotic consumption in 204 countries shows that between 2000 and 2018, the daily consumption of antibiotics increased by as much as 46% and this increase is the highest in Eastern Europe and Central Asia [68]. Based on the above data, periodontal bacteria resistance could not be generalized. For example, while in the case of *P. gingivalis* strains antibiotic resistance has not emerged, there have been observations of an increase in antibiotics (tetracyclines, macrolides, lincosamide, fluoroqinolones) minimal inhibitory concentrations and resistance transfer from related species of bacteria, which is a real threat [69].

## 5. Phages and Their Characteristics

A promising solution to the problem of both growing antibiotic resistance observed in bacteria and therapeutic failure in the treatment of periodontal disease with these antimicrobials may be bacteriophages (phages)—viruses that can recognize and destroy only the bacteria for which they are specific. Among the life cycles of bacteriophages, two of particular importance can be distinguished: the lytic cycle, leading to the destruction of the bacterial cell, and the lysogenic one. In the latter, the phage nucleic acid becomes integrated into the bacterial genome and may remain until the environmental conditions change to favor its release. In addition, there is also the phage life cycle called pseudolysogeny, in which the genetic material of bacteriophage forms an episome, as well as a condition called chronic infection [70]. Lytic phages (also known as virulent phages) are particularly recommended for therapeutic purposes because of their direct action against invasive bacteria and consequently a reduction of the bacterial population [71,72]. Lysogenic cycle phages (temperate bacteriophages) can be beneficial to the bacterial host by carrying genes that promote its pathogenicity, antibiotic resistance- or toxin-encoding genes, and can also interact with the human immune system in various ways [73]. Interestingly, there are reports of studies on the modification of temperate phages in order to adapt them to therapy [74].

It is assumed that each bacterial host has its own phage, which indicates a high probability of success in the search for and isolation of phages specific for pathogens constituting the etiological factor of periodontitis. Phages were known and applied before the introduction of antibiotics, they have not seen much development due to the relative comfort of antibiotic administration and usage [75]. Problems lie also in the necessity of targeted, specific therapy, and shortened activity/availability as well as activity of the phage particles in unfavorable conditions [76]. Several years ago it was claimed that, although bacteriophages might serve as a helpful antibacterial agent, the diversity of oral microbiota casts doubt on the usefulness of their implementation in periodontal treatment [77].

Recently, the importance of the composition of the natural microbiota of various areas of the human body, including the phages that inhabit them, has been clearly emphasized. The role of bacteria, viruses (including bacteriophages), fungi and protozoa naturally occurring in the human body, is highlighted in both health and disease [78]. Due to increasingly modern genetic-based methods, it has been also possible to gain knowledge about the composition of human oral microbiota [43,79].

Bacteriophages which are considered to be the most numerous “entity” in the human body [80] play a role not only in the elimination of bacteria but also modulate the response of the human immune system [81,82,83]. It has also been suggested that phages present in the mucus layer protect against pathogen invasion [84,85].

## 6. Phages and Their Contribution in Oral Microbiota

Phages have also been proven to be present in the oral cavity [85,86,87,88,89,90,91,92]. They may have a protective or etiopathogenetic contribution in the oral microbiota composition [93]. They may be found in dental plaque, saliva, oral washings [94,95,96,97]. It was proved that there are approximately 10^7^ viral-like particles (VLPs) per milligram of dental plaque [98]. Transcriptome analysis performed by Santiago-Rodriguez and co-workers revealed that out of all the viral reads from saliva samples collected from a cohort of healthy and diseased individuals, more than 90% of all tested samples were associated with bacteriophages [99]. In virome studies based on oral wash samples, it was described that a significant proportion of viruses are bacteriophages, both lytic and prophages (present in different niches) incorporated in the bacterial genomes, while a large number of viruses only affect a small number of patients [100]. However, there may potentially be more than 30 times more viruses in the oral cavity of a person than bacteria [93]. It is estimated that in the oral cavity (in the mucosa, dental plaque and saliva), about 2000 different phages can be found, specific to different species of bacteria, belonging to phyla such as *Firmicutes*, *Proteobacteria*, *Actinobacteria*, *Bacteroidetes* or *Fusobacteria* [101]. Among phages active against bacteria of the *Firmicutes*, the special importance of *Streptococcus*-specific phages is emphasized [102]. The presence of jumbo phage genomes (200–500 kbp), mainly on the surface of the tongue, while not in the intestines, is also of great importance [103]. The symbiosis of the microbiota naturally inhabits a specific part of the human body with its own cells/tissues and can result in many benefits for the host [104]. Bacteriophages can play an important role in controlling bacterial populations, and any disturbance of their proportion can lead to microbial dysbiosis, the importance of which is marked in the development of many diseases, including those in the oral cavity [93,104].

Interestingly, studies on the polymicrobial periodontal disease mouse model (occurring by oral infection by *P. gingivalis*, *T. denticola*, *T. forsythia*, *F. nucleatum*) based on metagenomics data presented a significant increase in viral diversity and content to infection when compared to a control, whereas a decrease was observed in bacterial diversity in infected mice [21]. This may suggest a meaningful role of phages in the development of periodontal disease.

Still little is known about the interactions between phages, bacteria and the host, but phages are believed to take an active role in the maintenance of eubiosis by controlling the growth of the biofilm [84]. Since knowledge about phages is still developing, authors often admit that only a small portion of the examined material was homologous to known viruses. This number for the study cited above equaled 0.16% [99]. The specificity of bacteriophages to bacterial cells is also accompanied by the lack of harmful effects on human cells. Furthermore, they may modulate the immune response [9,105,106]. Phage therapy is highly specific—it does not affect the host in any way, also it does not affect any pathogens other than the very one it is able to infect [107].

The above-mentioned study of total oral transcriptome evaluated differences between the status of healthy individuals and patients with periodontitis and revealed significantly more phage-related sequences in the former group, which may suggest an association between phages and eubiosis. A more detailed evaluation of the phage homology revealed that the major change occurred in *Firmicutes*, while for *Proteobacteria* the difference was statistically insignificant [99]. *Firmicutes* is the major phylum of Gram-positive bacteria. It is supposed that in the case of these bacteria in a natural environment phages play a rather protective role (most periopathogens belong to *Proteobacteria* phylum). For example, studies by Shlezinger et al. (2019) showed that phages active against *E. faecalis* in suspension or sustained release formulation applied to the root canal caused a change in microbiota composition: a decrease in the abundance of *Firmicutes* which corresponded with an increase in the relative abundance of *Proteobacteria* [86].

Due to the recent research, it is known that some bacteriophages, mostly temperate (coexisting with bacteria in prophage form), can have a harmful effect on the periodontium. For example, *Aggregatibacter* bacteriophages were proved to transfer antibiotic resistance genes (tetracycline resistance transposon) [108]. Interestingly, oral metagenome analysis showed a presence: *pblA* and *pblB* genes in the *Streptococcus mitis* SM1 phage genome [109]. The mentioned genes mediate the attachment of *S. mitis* to platelets. It is believed that the presence of the same bacteria in healthy and diseased individuals may be described by the modification of their properties and proteome due to inclusion in the structure of biofilm and exchange of metabolites and signaling molecules, but it is not excluded that viruses are responsible for such a change. Recently, Zhang et al. (2019) have described the *Siphoviridae*_29632 phage which is highly associated with severe outcomes in advanced periodontitis. Its prevalence was almost ten times higher in diseased than in healthy individuals [110]. Much attention has been paid to temperate phages specific to the serotype b (JP2) of *A. actinomycetemcomitans*, bacteria associated with the rapidly progressive form of periodontitis, affecting central incisors and first molars already in adolescents [111]. However, the described data indicate that the prophages encoded in the genomes of these bacteria are not directly related to the virulence factor associated only with periodontal disease. Since another study shows possible phage influence on the enhanced release of leukotoxin A, possible harmful effects of temperate phages on *A. actinomycetemcomitans* remains an open question [112].

## 7. Potential Phage Application in Periodontal Diseases

The use of lytic phages in therapy could apply to both periodontitis and peri-implantitis. In particular, it implies using phages active against bacterial biofilm. The potential strategy assumes the use of cocktails containing phages active against most selected known bacteria involved in the periodontitis/peri-implantitis, or modifying phages to increase their specificity against other bacterial strains and species [113]. It would almost certainly require the aforementioned mechanical therapy (in fact, every known periodontal treatment strategy includes professional debridement, due to the high resistance of biofilm to external environments).

The review by Szafrański and co-workers described potential new ways to utilize phages in periodontal therapy: using phages as specific eliminators of a given bacterial strain to examine the consequences of its absence in oral biofilm, and using phages or their lysins to enhance the action of antibiotic therapy and overcome bacterial resistance [114]. It is postulated to use phages in prophylaxis to protect against bacterial colonization, similarly to the methods already used in the food industry [114].

The complex structure of biofilm is resistant to most antibiotics, which do not have the ability to penetrate this structure. It seems that applying phages in therapy may be useful in biofilm control [115]. Phages may play a complicated role in the biofilm, including its destruction [116]. It is indicated that phages have the ability to access dense biofilm and weaken its structure through spreading the tightly packed neighboring cells [117]. Previous studies show that phages are also specific to bacteria forming biofilm, which are the etiological factor of periodontitis. Bacteria which play an important role in the development of oral biofilm is *Fusobacterium nucleatum* [22]. It is also a putatively important pathogen in the aetiology of the cancers of the digestive tract, including the oral cavity [118]. Kabwe et al. (2019) have identified and studied FNU1, a lytic phage specific to *F. nucleatum* which administration on the biofilms containing these bacteria resulted in a 70% reduction of *F. nucleatum* biomass [89].

Common biofilm-associated diseases affecting tissues surrounding implants are mucositis and peri-implantitis [19] which may affect more than 50% of dental implants [19,119]. In endodontics, however, eradication of the biofilm is required, while in periodontology, change from a dysbiotic biofilm to a symbiotic biofilm is only possible, as eradication of an individual bacterium is observed. Therefore, coating dental implants with bacteriophages may be a solution [120]. Interestingly, there is the possibility that a phage peptide (using phage display method) binds to the surface of zirconia which may suggest a possible interference (by electrostatic interaction) with biofilms that cause peri-implantitis [117,121].

The majority of the available studies concerning peri-implantitis concentrate on *Enterococcus faecalis*, a species often isolated from the root canal system of teeth with reported complications of endodontic treatment. This bacterium is often resistant to the vast majority of not only antibiotics, but even chemotherapeutics, and is one of the reasons for which, chlorhexidine cannot be used for root canal rinsing. Instead, sodium hypochlorite (NaOCl) is used, which may result in numerous complications for the patient (such as emphysema). In a study by Bhardwaj et al. (2020), an isolated phage belonging to *Siphoviridae*, specific to *E. faecalis*, caused a significant reduction (5 log CFU/mL) of the biofilm 24 h after topical application [122]. Interestingly, multidrug-resistant *E. fecalis* in an ex vivo root canal infection model was also effectively eliminated after irrigation with the use of vB_ZEFP phage suspension and with NaOCl and phage combined treatment [123]. These results support the earlier report by Tinoco et al. (2016), who genetically modified the phage specific to *E. faecalis* [124]. Phage ØEf11 was enriched with open reading frames of another phage (ØFl11c) to increase its affinity to various *E. faecalis* strains and the determinant repressor fragment was deleted to prevent the phage from entering the lysogenic phase such a precaution is validated and commonly used in phage engineering, since it limits the danger of unpredictable gene insertions and mutations in the bacterial genome [124]. For the same reason, the control promoter of the lytic cycle was deleted, followed by the insertion of a nisin-induced promoter, so that the activation of the phage would start in the presence of bacteriocin [124]. The obtained phage ØEf11/ØFl11c(Δ36)PnisA proved to be very effective in the elimination of bacteria. The engineered virus existed only in the lytic phase (resulting in the destruction of bacterial cells) and was resistant to suppression with the *CI* gene. It proved to have a wider lytic spectrum in comparison to wild phages, and required a trigger (nisin) to activate. The mentioned phage caused 10–100 fold elimination of *E. faecalis* strains, both sensitive to vancomycin or vancomycin-resistant (JH2-2 and V583 strains, respectively) [124]. As has been presented above engineered/modified phages are a branch of the research on bacteriophages that opens up new possibilities, and potentially helps to overcome most problems related to phage therapy [125]. Phages can be modified by means of altering their capsid or modification of the genome [126], either with the use of naturally emerging or artificially created molecules (bioconjugation) [127] as well as tail fiber engineering [128] or lysin [129]. Interestingly, a phage display method can be used to identify the inhibitor of metalloprotease like enzyme—karilysin—synthetized by *T. forsythia* which may be helpful in the modification of pathogenicity of this bacterium, which causes an advanced form of periodontal disease [130].

Furthermore, endolysin-bacteriophage-encoded peptidoglycan hydrolases [131,132] have an antimicrobial potential, consisting of, inter alia, a lack of resistance mechanism ability to reduce biofilm, the potential for engineering [133], as well as rapid bactericidal activity. The properties mentioned above indicate that these enzymes may be successfully used in the treatment of periodontitis. Their activity is also observed against multidrug-resistant bacteria [134]. The above-mentioned properties may help to omit limitations in phage therapy presented in the next section. For example, interesting findings presented by Nilebäck et al. (2019) who used silk coatings fused with endolysin or Dispersin B and observed the reduced formation of *S. aureus* biofilm by the weakness of their adhesion [135].

The type of formulation of the phage preparation may be crucial from a therapeutic point of view. Using phages in a nonconventional form or composition, as with the thermoreversible sustained release formulation, was considered as a possible method to help prolong phage activity [86]. When poloxamer P407, which is a nonanionic emulsifying agent with a viscosity change dependent on temperature (low at 4 °C), was added to a phage cocktail active against *E. faecalis* in a solution with a titer of ~10^9^ PFU/mL, prolonged phage activity even over the course of one month was observed. As a result of the application of sustained release phage formulation in vivo, a 99% reduction in *E. faecalis* count was observed (whereas phage cocktail suspension reduced 95% of bacteria in root canal infection in vivo). Poloxamer has also been proven to be nontoxic to mouse macrophages. Gel consistency is the feature which causes the formulation with phages to be considered a possible ideal intracanal therapy especially because of prolonged activity at the infection site. Application of phages-loaded alginate-nanohydroxyapatite hydrogel for local tissue regeneration and infection prevention (*E. faecalis* colonization) and control was proved to be effective [136] which may suggest the effective application of similar formulation in peri-implantitis. It is also suggested that this means of phage application may prevent reinfection. Interesting observations were made by Wolfoviz-Zilberman et al. (2021) who evaluated the effect of the *Streptococcus mutans* SMHBZ8 phage in the prevention of carious lesions in vitro and in vivo [88]. They used phage in a formulation with polymer varnish (in a 2:1 ratio) which was much more effective in preventing *S. mutans* infection than using phage suspension. A hydroxypropyl-cellulose-based formulation containing the *S. mutans* phage in vivo was also suggested to be examined [88]. Other delivery routes which may be considered in periodontal disease are, among others: chewing gum, polycaprolactone/collagen I nanofibers, syrup [88], toothpaste, tablets [137] as well as liquid mouthwash. Advances in medical carriers allow the production of stable mRNA vaccines, which would be even more helpful to deliver stable phage particles.

## 8. Challenges in Phage Therapy

It should be pointed out that potential phage therapy has its pros and cons (which were summarized in Table 2). The following advantages should be emphasized [138]: specificity towards target bacteria without a harmful effect for eukaryotic cells [139]; ability to self-replicate at the infection site. The therapy disadvantages [110] may be: the possibility to acquire bacterial resistance to phages; the potential to induce an immune response, despite the mentioned non-toxicity phages are recognized by the immune system as foreign particles, and bacterial lysis may lead to an increased concentration of endotoxins.

Phage application may be associated with the acquisition of resistance by bacteria to the used phages and result in a lack of efficiency by the phage. Its probability may be observed, in particular, in the case of oral microbiota which is composed of different bacteria from different niches and horizontal gene transfer resulting in phage resistance gene acquisition [147]. Using cocktails may eliminate the possibility of developing phage resistance in bacteria and also may cause improvement in their lytic spectrum [148,149]. Furthermore, using genetically modified phages may be a solution allows the phage host range to be expanded [150].

As was mentioned in Section 6, the oral phageome is mostly composed of temperate phages. This type of phage is not recommended for therapeutic purposes [145]. However, using genetic modifications it is possible to remove genes responsible for lysogeny [151]. Because phages are immunogenic they are neutralized by the immune system even when they are administered per os [124,125,126,127,128,129,130,131,132,133,134,135,136,137,138,139,140,141,142,143,144,145,146,147,148,149,150,151,152,153]. Long circulating phage mutants with enhanced survival after administration may solve problems connected with loss of phage titer after their application [154]. There are also possibilities to reduce phage toxicity and/or immunogenicity [155] as well as to obtain modified phages with improved activity against bacterial biofilms [156].

There are no data regarding phage application against periodontitis and/or peri-implantitis in an animal model. This generates difficulties in translating observations in vitro to in vivo conditions. This is further exacerbated by periodontal disease having a multifactorial basis and that it is difficult to successfully recreate the conditions prevailing in the oral cavity (including the complete composition of the microbiota) in vitro which does not facilitate prompt clinical phage application.

Much requires explanation regarding the dynamics of phage colonization in oral microbiota. It is noteworthy that phage and antibiotic pharmacokinetics are entirely different: phage amplification on target bacterial strains causes an increase in active phage particles following phage administration white antibiotic concentration decreases with time. Therefore, it is difficult to establish an optimal therapeutic phage dosage [157]. A number of factors determine the potential success of phage therapy in periodontal disease: phage interactions with oral microbiota, progress in phage pharmacokinetics, host immune response to administered phages, etc. [141,142,158,159]. Bacteria that are components of the red complex of periopathogens are difficult to cultivate, especially anaerobic bacteria, which makes the work difficult, limiting the possibilities of searching for and isolating phages specific for these pathogens. Moreover, the polymicrobial character of periodontal disease should be taken into consideration when phage preparation intended to prevent or treat periodontitis is being developed.

The high diversity and variability of bacterial strains recognized as periopathogens (sixty-two in the case of *Porphyromonas gingivalis*) [160] cast doubt on both the effectivity and economic reasonableness of the use of phage cocktails or modifying phages, even considering those three pathogens mentioned above included in (according to the former concept of periodontitis) the red complex of bacteria most associated with the disease. Instead, attention should be paid to either the most harmful putative strains and attempts made to compose a mixture of phages directed towards those strains, or each case of treatment should require a personalized design and approach. The latter strategy is possible due to the fact that in periodontal diseases infection has a chronic course and does not result in the quick perfusion of bacteria to the bloodstream and colonizing distant organs. One can utilize wild phages (existing naturally in oral microbiota), phages modified by means of genetic engineering, or lysins-proteins encoded in phage genomes, causing bacterial cell wall lysis, enabling the release of phage progeny amplified inside the bacterial cell [106].

There is a rationale for further studies and elucidation of the structure, functions and potential use of phages, not only in periodontal therapy, but also in other medical and nonmedical disciplines [161], since there is a possibility to achieve major breakthroughs or advances in prophylaxis and/or treatment of several diseases and conditions currently affecting the health and well-being of the human population.

## 9. Conclusions

As pointed out AMR is a growing challenge to our civilization and new tools to combat this threat to global health are urgently needed. Current data suggest that phage therapy offers such a tool potentially applicable in some diseases of the oral cavity. As of today, it appears that phage therapy offers an interesting perspective for the treatment of periodontal disease which is prompted by the current lack of targeted, highly effective and readily available antibacterial agents applicable in that condition. However, further progress in our understanding of the interactions of phages with the oral microbiome is needed to accomplish that goal.

## Figures and Tables

**Table 2:** Pros and cons of using phages in periodontal diseases.

Pros		Cons	
Property	Consequence	Property	Consequence
Host specificity for recognized pathogens only [9]	Target only pathogens against which they are active. Natural microbiota not affected	Short activity/availability [140,141,142]	Inactivated in the human body, therefore, the therapeutic effect may be weak
Presence in the environment (including oral cavity)	High probability of success in the search for and isolation of phages	Loss activity of the phage particles in unfavorable conditions [143]	Weak therapeutic effect
Possible modulation the response of the human immune system [74,106]	Phages present in the mucus layer protect against pathogen invasion	Possible development of phage resistance [144]	Lack of therapeutic effect
Amplification at the infection site	High phage titer is possible to achieve with resulting eradication of the pathogen	Temperate phages may carry toxins and/or antibiotic resistance genes [111,112]	Therapeutic phages should be devoid of genes coding for integrases, antibiotic resistance as well as toxins in their genomes [145]
Lack of serious side effects [9]	Good tolerability		
Safe for immunocompromised patients [146]	May be applied in immunocompromised patients		
Proven activity against periodontal biofilm [87,88,89,123]	Potential applicability in periodontal disease		
Phage cocktails available	Reducation of bacterial resistance and wider spectrum of activity		
Various forms of phage application assuring their sustained release [8,88]	Assuring efficient phage concentration at the site of infection and extending phage persistence thus prolonging the possible therapeutic action		

## Data Availability

Not applicable.
